# The Effect of Psychology Objective Structured Clinical Examination Scenarios Presentation Order on Students Autonomic Stress Response

**DOI:** 10.3389/fpsyg.2021.622102

**Published:** 2021-03-24

**Authors:** Alberto Bellido-Esteban, Ana Isabel Beltrán-Velasco, Pablo Ruisoto-Palomera, Pantelis T. Nikolaidis, Beat Knechtle, Vicente Javier Clemente-Suárez

**Affiliations:** ^1^Faculty of Psychology, Universidad Europea de Madrid, Madrid, Spain; ^2^Department of Education, University of Nebrija, Madrid, Spain; ^3^Department of Health Sciences, Public University of Navarre, Pamplona, Spain; ^4^Department of Physical and Cultural Education, Hellenic Army Academy, Athens, Greece; ^5^Exercise Physiology Laboratory, Nikaia, Greece; ^6^Institute of Primary Care, University of Zurich, Zurich, Switzerland; ^7^Faculty of Sport Sciences, Universidad Europea de Madrid, Madrid, Spain; ^8^Grupo de Investigación en Cultura, Educación y Sociedad, Universidad de la Costa, Barranquilla, Colombia

**Keywords:** OSCE, autonomic stress response, executive functions, complexity, undergraduate

## Abstract

The objective structured clinical examination (OSCE) is a method for assessment clinical competencies and skills. However, there is a need to improve its design in psychology programs. The aim of this study was to analyze the effect of the different scenario's presentation order with different complexity/difficulty on the autonomic stress response of undergraduate students undergoing a Psychology OSCE. A total of 32 students of Psychology Bachelor's Degree (23.4 ± 2.5 years) were randomly selected and assigned to two OSCE scenarios of different complexity. While undergoing the scenarios, participants heart rate variability was analyzed as an indicator of participant's stress autonomic response. Results indicate that the order of presentation of different complexity/difficulty scenarios affects the autonomic stress response of undergraduate Psychology students undergoing an OSCE. Students who underwent the high-complexity scenario (difficult) first, reported significantly higher autonomic stress response than students who began the OSCE with the low-complexity scenario (easy). Highly complex or difficult scenarios require good executive functions or cognitive control, very sensitive to autonomic stress responses. Therefore, OSCE design will benefit from placing easy scenarios first.

## Introduction

Clinical simulation scenarios provide to the university students the clinical experience necessary for their professional future (Munroe et al., 2016; Sobh et al., 2017a,b). Therefore, the use of these scenarios is being implemented powerfully in universities that offer degrees in the health sciences area, becoming an indispensable tool in the teaching-learning process (Bradley, 2006; Borggreve et al., 2017).

Specifically, in the Psychology degree, interaction with simulated patients has become a learning and assessment tool that brings practical knowledge to the theory that students have learned during the degree (Crego et al., 2016). In this line, Objective Structured Clinical Evaluation (OSCE) is a methodology used in Psychology degree in the last year of the degree to prove students in a controlled clinical context with no real risks for patients (Clemente-Suárez et al., 2018). In this environment, each student must attend a simulated patient in a clinical session, showing previous research that this simulation context produces a large anxiogenic response in students (Starcke et al., 2016; Beltrán-Velasco et al., 2018).

This anxiogenic response could negatively affect the ability of each student to efficiently cope with the acquisition of the learning process (Unsworth et al., 2014; Starcke et al., 2016). It is known that a high sympathetic modulation has a direct relationship with alterations in the correct communication between neurons of the pre-frontal cortex of the brain (Delgado-Moreno et al., 2019). A negative correlation has been observed between the hyperactivation of sympathetic nervous system and cortical arousal, information processing and memory (Chan et al., 2008). When the organism interprets a stimulus as a possible threat or stressor, the sympathetic nervous system and the hypothalamic-pituitary-adrenal axis are activated, increasing production of cortisol, adrenaline, and noradrenaline (Morgan et al., 2006; Hood et al., 2015). Although these actions are not conscious, they would induce both, structural and functional changes in neurons, producing a general decrease in the executive function's performance: low emotional regulation, attentional and memory difficulties, etc. (Kane and Engle, 2002; Clemente-Suárez and Robles-Pérez, 2013).

The analysis of high anxiety response in this context has been conducted by previous research measuring the autonomic modulation by the Heart Rate Variability (HRV) (Clemente-Suárez, 2015; Clemente-Suárez et al., 2017; Beltrán-Velasco et al., 2019) and psychological factors like personality, cognitive flexibility, life purpose, perceived stress, among other (Giles et al., 2015; Sánchez-Conde et al., 2019). But the relation of the stress response and the academic performance of Psychology degree students is already poor know. Then, in order to improve this knowledge that could allow to design better practical scenarios in Psychology degree, as is the OSCE, we conducted the present research. Therefore, the aim of this study was to analyze the effect of the scenario's presentation order with different complexity/difficulty on the autonomic stress response of undergraduate students undergoing a Psychology OSCE. We hypothesized that high to low complexity scenarios presentation order would produce a higher autonomic stress response.

## Materials and Methods

### Participants

The sample was composed by a total of 32 students of the final course of Psychology degree (12.5% men and 87.5% women). Age ranged from 21 to 30 years old (*M* = 23.75; *SD* = 2.51). This study was approved by the Ethics Committee of the European University of Madrid (CIPI/18/074) and was conducted according to the principles expressed in the Declaration of Helsinki. Written informed consent was obtained from all participants. All students are novices in clinical practice with no prior experience. The evaluation was conducted at the simulated hospital located in the Faculty of Health Sciences of the European University of Madrid (Spain).

### Measures and Instruments

Autonomic stress response. The following HRV parameters were recorded through the Polar V800 heart rate monitor (Polar, Kempele, Finland) and analyzed based on R-R heartbeat intervals using the Kubios HRV software (version 2.0, Biosignal Analysis and Medical Imaging Group, University of Kuopio, Finland) (Malik et al., 1996; Ramírez-Adrados et al., 2020a). In particular, the following parameters were assessed: percentage of differences between normal adjacent RR intervals >50 ms [PNNx50 (ms)]; square root of the average of the sum of the differences squared between normal adjacent [RMSSD (ms)]; and sensitivity of the short and long-term variability, respectively, of the non-linear Specter of the HRV [SD1 (ms) and SD2 ms)].

### Design and Procedure

HRV parameters [LF (nu), HF (nu), PNN50 (No.), RMSSD (ms), SD1 (ms), and SD2 (ms)], as indicators or autonomic stress response, were collected at the beginning, in the middle and at the end of the OSCE using the Polar V800 Heart Rate Monitor (Polar, Kempele, Finland) was placed in participant's wrist 5 min before OSCE start. Participants registered for the study were randomly selected from the class roster of Adult Psychological Treatment and Childhood and Adolescence Psychological Treatment. Then, participants were randomly assigned to one of the two conditions. Each condition differs in the order of presentation for the two scenarios included in the OSCE. Each student spent 15 min in each scenario. All participants received the same 1 min oral description of the content of each scenario beforehand. The Polar device was removed from participants wrist 5 min after the second and last scenario finished. All participants had the same “clinical” training.

The two 15 min scenarios were carefully designed for differ in complexity based on the number of patients involved, severity and chronicity of the problems. The high-complexity scenario (more difficult) consisted of two parents with a 10 year history of serious men harassment and aggression outburst toward the women aggravated with drug problems. The session included interruptions to the student and a non-collaborative attitude. The low-complexity scenario (easier) consisted on one adult woman with mild anxiety and depressive symptoms after a sentimental breakup. No interruptions to the students were included during the session and her attitude was collaborative. All scenarios were performed in experimental room with a one-way mirror, decorated as a classical therapeutic room for ecological validity and the same professional actors (Sobh et al., 2017a) for representing the roles for the two scenarios for each student.

### Statistical Analysis

All statistical analyses were carried out using the IBM Statistical Package for the Social Sciences (SPSS) software version 21.0 for Windows. Descriptive statistics for the variables (M, SD) were analyzed. Then, multiple one-way analyses of variance (ANOVA) were performed to evaluate the effect of the order of presentation of the two scenarios (high and low complexity/difficulty) on the 6 parameters of HRV. Levene's and Shapiro Wilk's tests were used to test assumption of normality and equality of variances. Effect size was calculated using partial etha squared (η^2^). RMMSD and PNN50 were logarithmically transformed to meet normality. The level of significance was set at 0.05.

## Results

### Perceived Complexity/Difficulty of Scenarios

Participants' evaluation of scenarios difficulty confirmed that the two scenarios significantly differ in complexity based on their rates on a scale ranging from 0 to 10, where 10 represent the highest level of complexity or difficulty and 0 the lowest. High-complexity scenario was rated with *M* = 8.15, *SD* = 1.09; while low-complexity scenario was rated at 4.07, *SD* = 1.01. Differences in perceived difficulty or complexity were statistically significant, *t*_32_ = 3.72; *p* < 0.001.

### Effect of Order of Presentation on HRV Parameters (Autonomic Stress Response)

**Figure 1:**
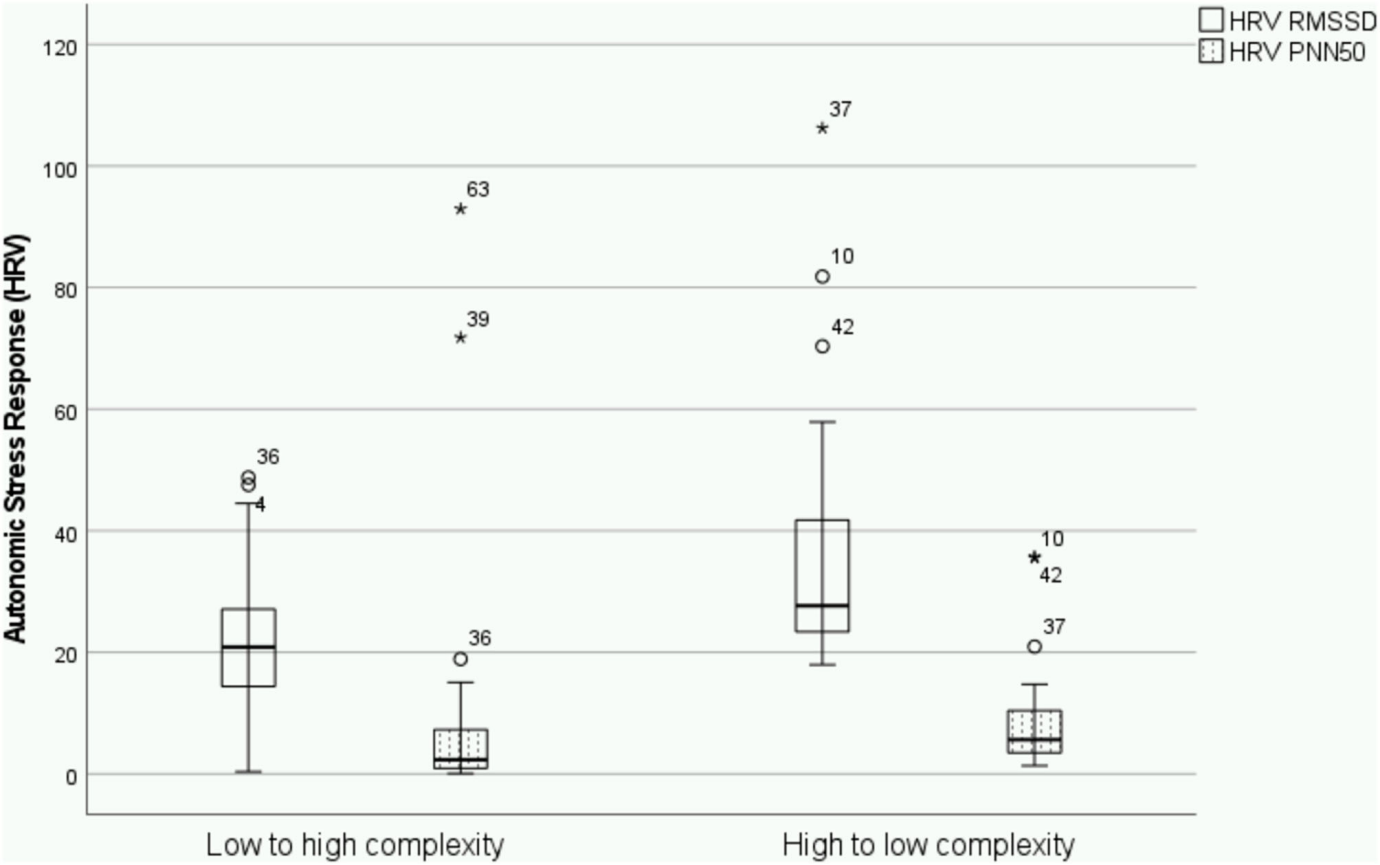
Box plot displaying differences on the effect of the order of presentation of high and low complexity/difficulty scenarios on RMMSD and PNN50 as measures of autonomic stress response. The asterisk in the boxplot represents extreme values (points at a greater distance from the median than 1.5 times).

**Figure 2:**
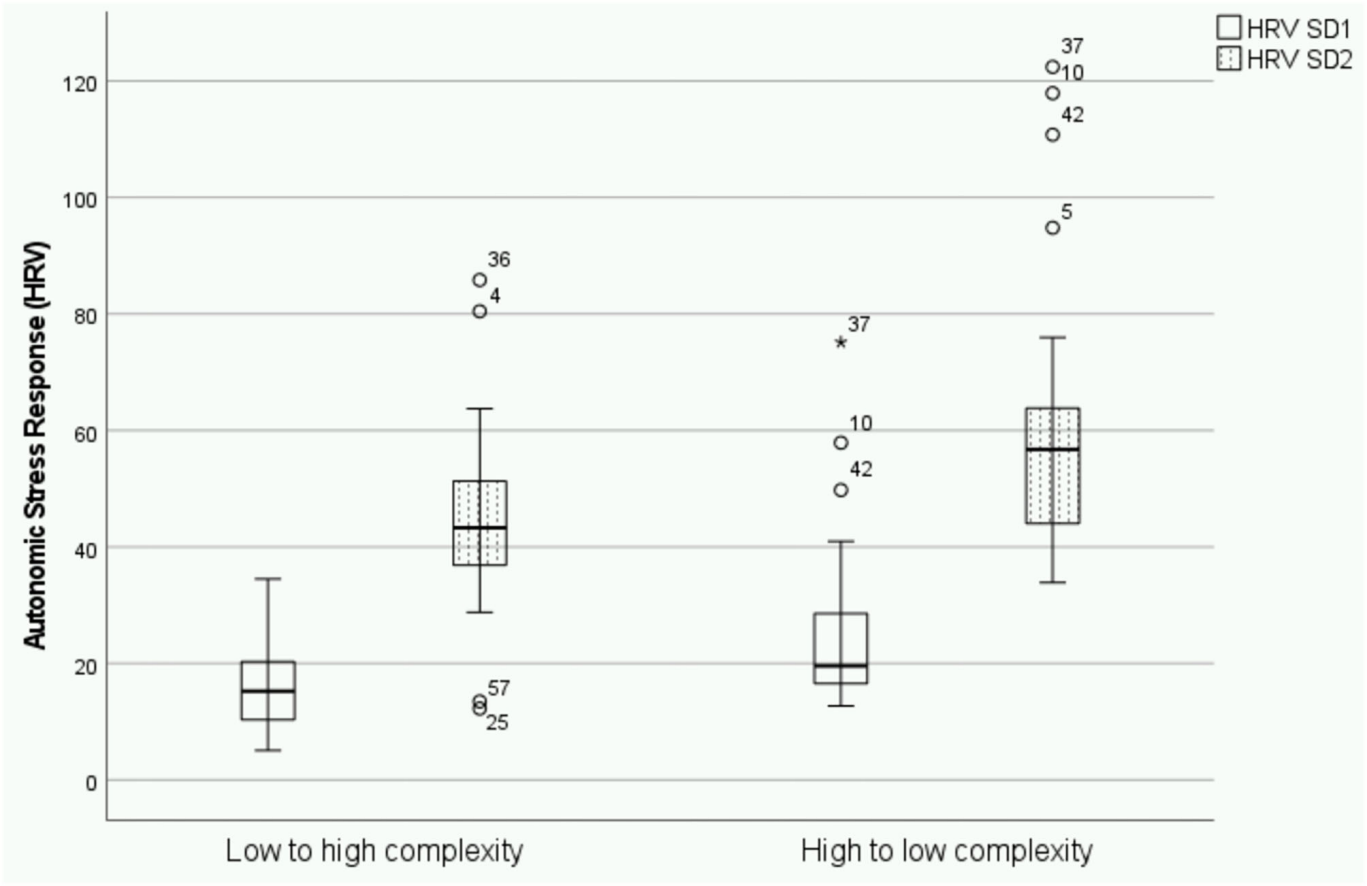
Box plot displaying differences on the effect of the order of presentation of high and low complexity/difficulty scenarios on SD1 and SD2 as measures of autonomic stress response. The asterisk in the boxplot represents extreme values (points at a greater distance from the median than 1.5 times).

Results of ANOVA showed a consistent significant effect of the order of presentation of scenarios for all HRV parameters of autonomic stress response analyzed. Autonomic stress response during OSCE was significantly higher for those participants presented first with the high-complexity/difficulty scenario and then with the low-complexity/difficulty scenario than participants expose to the alternative order. Moreover, the effect size was medium for all 4 HRV parameters (Table 1). Interestingly, no effect of type of scenario was found (Figures 1, 2): PNN50 [*F*_(3,1)_ = 0.453; *p* = 0.504], RMMSSD [*F*_(3,1)_ = 1,170; *p* = 0.682], SD1 [*F*_(3,1)_ = 0.551; *p* = 0.461], SD2 [*F*_(3,1)_ = 0.007; *p* = 0.932]; and therefore, no interaction was found between the type of case and the order of presentation: PNN50 [*F*_(3,1)_ = 2.697; *p* = 0.106], RMMSSD [*F*_(3,1)_ = 0.004; *p* = 0.948], SD1 [*F*_(3,1)_ = 1.861; *p* = 0.178] y SD2 [*F*_(3,1)_ = 1.560; *p* = 0.217].

**Table 1:** Descriptive statistics (M ± SD) in the average autonomic stress response of psychology undergraduate students during the OSCE.

	**Condition**						
	**Low to high complexity**		**High to low complexity**				
**HRV**	**Low**	**High**	**High**	**Low**	**HRV**	**Low**	**High**
PNN50 (No.)	0.27 ±.55	0.58 ± 0.65	0.72 ± 0.38	0.85 ± 0.36	5.44	*p =* 0.011	0.111
RMSSD (ms)	1.29 ± 0.18	1.25 ± 0.46	1.48 ± 0.18	1.52 ± 0.22	9.42	*p =* 0.003	0.136
SD1 (ms)	14.95 ± 6.34	21.7 ± 13.29	24.3 ± 12.6	26.3 ± 17.5	4.73	*p =* 0.033	0.128
SD2 (ms)	41.18 ± 13.56	47.77 ± 15.29	58.71 ± 23.93	64.45 ± 4.82	12	*p =* 0.001	0.171

*PNN50, the proportion of NN50 divided by total number of NNs; RMMSSD, Square root of the mean of the sum of the squared differences between adjacent normal R–R intervals; SD1, standard deviations of the scattergram 1; SD2, standard deviations of the scattergram 2; n.u. Normalized unit*.

## Discussion

The main goal of this study was to analyze the effect of the scenario's presentation order with different complexity/difficulty on the autonomic stress response of undergraduate students undergoing a Psychology OSCE. The main finding was that the autonomic stress response during the OSCE was significantly higher for those participants presented first with the high-complexity/difficulty scenario and then the low-complexity/difficulty scenario than participants expose to the alternative order. According to these results the initial hypothesis was complied.

We found how the OSCE produced in student a large stress response as the low scores on HRV parameters showed. This result was related to a large sympathetic nervous system activation due to the exposition to an unknown and uncertain context that is perceived by students as a threatening and uncontrollable situation (Sánchez-Conde et al., 2019). The increase in the sympathetic modulation prepares students to respond for the possible uncertainties of the academic scenario. This response could be elicited by real threat, or simply by the perception of uncontrollability or threat (Belinchón-deMiguel et al., 2019). Then, we can see how the OSCE is a source of novelty and uncontrollability, which has provoked a high sympathetic response in the students to face this new situation. This autonomic response was also evaluated in other stressful scenarios since extreme sport events, as ultraendurance mountain races (Belinchon-Demiguel and Clemente-Suárez, 2018; Tornero-Aguilera and Clemente-Suárez, 2018), to military underground operations (Hormeño-Holgado and Clemente-Suárez, 2019a), special operations interventions (Hormeño-Holgado and Clemente-Suárez, 2019b), military aircraft combat maneuvers (Vicente-Rodriguez and Clemente-Suárez, 2020), and helicopters crews in action (Appelhans and Luecken, 2006). This information reveals how the body's defense systems are activated in the same way regardless of the nature of the stressor stimulus, causing a similar sympathetic response in psychology students and military or ultraendurance athletes.

The negative impact of high autonomic stress response on performance has been extensively studied (Belinchón-deMiguel et al., 2019). However, to our knowledge, this is the first study to systematically examine the effect of the order of presentation of scenarios of high and low levels of complexity or difficulty in the context of OSCE. The large autonomic stress response evaluated produce deleterious effects on executive functions, essentials for successfully dealing with complex problems and emotional regulation (Soliemanifar et al., 2018). In this line, individual differences like some personality factors or previous life experiences would impact on the ability to modulate the stress response making some people more vulnerable (Saravanan and Wilks, 2014; Hood et al., 2015). In the confrontation of a OSCE scenario in which the students' clinical abilities are evaluated is expected an autonomous sympathetic activation, as previous studies reported (Sánchez-Conde et al., 2019), causing an increase in blood pressure and heart rate, the liberation of cortisol that would negatively affect neurons communication in pre-frontal cortex, fact that could negatively affect cortical process as memory, attention, planning, decision making or reflective thinking (Diamond, 2013; Starcke et al., 2016; Beltrán-Velasco et al., 2020; Mendoza-Castejón and Clemente-Suárez, 2020; Mendoza-Castejon et al., 2020; Ruisoto et al., 2021). Then, to minimize the negatives effect of stress in students, the complex or difficult scenarios must be place after easier ones. These results are consistent with the well-known habituation effect, by which students experience a reduction in their autonomic stress levels during the easier scenario, preventing facing later complex scenarios with lower levels of autonomic stress response and, therefore, a lower risk of impaired executive functions.

The control of the autonomic stress response could be also applied to other academic context like dissertation or clinical stays, context in where students also present a large autonomic stress response (Ramírez-Adrados et al., 2020b; Redondo-Flórez et al., 2020, 2021; Beltrán-Velasco et al., 2021; Clemente-Suarez et al., 2021). This uncontrolled response could result in a negative action of students, negatively having repercussions in their academic performance and finally in their learning process.

### Limitations of the Study and Future Research Lines

The principal limitation of the present research was the small sample size. The difficulty to recruit students in this complex scenario limited a large sample in the present research. Also, no measures of amylase or cortisol stress hormones limited the hormonal stress response analysis in the present research. This limitation was subject to a lack of enough technological and financial resources. These parameters could be used in future research to better understand of the psychophysiological stress response of students.

### Practical Applications

The autonomic response is directly associated with variations in HRV parameters that are objectively measurable. The use HRV in real time during OSCE could be a useful tool to increase students' performance. This information could help students to control the stress response using biofeedback instrument, as well-could help teachers to improve the evaluation process and the design of these professional simulation environments. In higher education, students could benefit from the use of these instruments to face the evaluation in simulated environments, being able to obtain an objective measure of their stress levels. This will allow the conscious use of strategies and skills to reduce distress and improve global cognitive functioning. Moreover, the stress response monitoring of students carried out in this study allows teachers to design OSCE more adapted to the students, limiting the effect of the stress response on the student, allowing a better performance of these.

## Conclusions

The order of presentation of different complexity/difficulty scenarios affects the autonomic stress response of undergraduate Psychology students undergoing an OSCE. Students who underwent the high-complexity scenario (difficult) first, reported significantly higher autonomic stress response than students who began the OSCE with the low-complexity scenario (easy). Highly complex or difficult scenarios require good executive functions or cognitive control, very sensitive to autonomic stress responses. Therefore, OSCE design will benefit from placing easy scenarios first.

## Data Availability Statement

The raw data supporting the conclusions of this article will be made available by the authors, without undue reservation.

## Ethics Statement

The studies involving human participants were reviewed and approved by Comité de Ética e Investigación de la Universidad Europea. The patients/participants provided their written informed consent to participate in this study.

## Author Contributions

AB-E collected the data and prepare the original draft of the paper. AB-V collected the data and contribute to the elaboration of tables and figures. PR-P conducted the statistical analysis and reviewed and edited of the final version. PN, BK, and VC-S conceived and designed the study. VC-S also performed the formal analysis, data curation, and reviewed and edited of the final version of the paper. All authors reviewed and agree with the final version of the manuscript.

## Conflict of Interest

The authors declare that the research was conducted in the absence of any commercial or financial relationships that could be construed as a potential conflict of interest.
